# THE ESSENTIAL ROLE OF PAID CAREGIVERS IN HOME-BASED DEMENTIA CARE

**DOI:** 10.1093/geroni/igad104.1196

**Published:** 2023-12-21

**Authors:** Jennifer Reckrey, Joanne Spetz

## Abstract

Paid caregivers (e.g., home health aides, other home care workers) provide essential support to the rapidly growing number of people with dementia living in the community. This symposium presents innovative findings on the role of paid caregivers in home-based dementia care though exploration of the clinical, family, and care-delivery contexts in which paid caregivers work. First, Reckrey et al describe the scope of paid caregiving in dementia using nationally representative data examining factors associated with receipt of paid care among older adults with overlapping dementia and non-dementia serious illness. Next, we examine mechanisms of delivering paid care: Osakwe et al explore paid caregiver perceptions about the use of telehealth in home health and identify paid caregivers concerns about routine use of this technology in care of people with dementia, and Gordon-Wexler et al explore family caregiver perspectives on navigating Medicare and Medicaid insurance for people with advanced dementia and the challenges they experience coordinating paid care. Finally, we explore how paid caregivers work with other care team members. Quach et al discuss multidisciplinary perspectives about how paid caregivers support person-centered and relationship-centered care to family dementia caregivers, while Fabius et al examine information sharing practices within home care agencies and describe differences and similarities in information sharing needs for people with and without dementia. Taken together, these presentations highlight the important ways that paid caregivers work with providers and families within the care delivery system and identify ways to better support and integrate these essential workers into dementia care teams. This is a Paid Caregiving Interest Group Sponsored Symposium.

